# Highly differentiated T cells link systemic and vascular inflammation in a mouse model of recurrent psoriasis

**DOI:** 10.3389/fimmu.2025.1574455

**Published:** 2025-06-17

**Authors:** Fabio Casciano, Paolo Severi, Laura Marongiu, Anna Caproni, Chiara Terranova, Alex Spitilli, Davide Ferrari, Chiara Ruzza, Peggy Marconi, Paola Rizzo, Francesca Granucci, Paola Secchiero, Eva Reali

**Affiliations:** ^1^ Department of Environmental and Prevention Sciences and LTTA Centre, University of Ferrara, Ferrara, Italy; ^2^ Department of Translational Medicine, University of Ferrara, Ferrara, Italy; ^3^ Department of Biotechnology and Biosciences, University of Milano-Bicocca, Milan, Italy; ^4^ Department of Life Sciences and Biotechnology, University of Ferrara, Ferrara, Italy; ^5^ Department of Neurosciences and Rehabilitation, University of Ferrara, Ferrara, Italy; ^6^ Department of Translational Medicine and LTTA Centre, University of Ferrara, Ferrara, Italy; ^7^ Maria Cecilia Hospital, GVM Care & Research, Cotignola, Italy

**Keywords:** T cells, mouse model of recurrent psoriasis, endothelial dysfunction, vascular inflammation, cardiovascular comorbidities

## Abstract

Psoriasis is a chronic inflammatory skin-disease associated with cardiovascular comorbidities. In patients, T cells with a skin-primed phenotype are expanded in peripheral blood, indicating a role for skin to blood T cell recirculation in the development of systemic comorbidities. Here, we aimed to investigate (i) the establishment of CD4^+^ and CD8^+^ T cell memory, (ii) the accumulation of activated and terminally differentiated T cells, and (iii) the potential link with vascular inflammation, in a mouse model of recurrent psoriasis. The results revealed systemic accumulation of memory T cells in the mouse model and similar results in patients with psoriatic disease. Recurrent psoriasis-like condition in mice also induced increased activation of memory T cells, augmented frequencies of CXCR3^+^4-1BB^+^ and PD-1^+^TIM-3^+^ CD4^+^ T cells as well as CD8^+^ T cells with a highly differentiated phenotype. Notably, parallel analysis in aorta samples revealed upregulation of endothelial dysfunction (*Icam1, Vcam1*) and vascular inflammation markers (*Ccl2, Olr1*), together with a trend towards increased expression of the CXCR3 ligand, *Cxcl10*. Importantly CXCR3^+^LFA-1^+^ CD4^+^ and CD8^+^ T cell effectors were markedly enhanced at systemic level, thus providing insights into the mechanistic link between highly differentiated T cells, endothelial dysfunction and vascular inflammation.

## Introduction

Psoriasis is a chronic recurrent inflammatory disease associated with joint manifestations and systemic comorbidities, including atherosclerosis and cardiovascular diseases (1–3). The immunopathogenic mechanisms of cutaneous psoriasis have been extensively studied, however the mechanisms linking skin inflammation to extracutaneous manifestations remain poorly understood (4–6). Emerging evidence suggests that skin-primed memory CD4^+^ and CD8^+^ T cells, amplified and activated within psoriatic lesions, may circulate contributing to the systemic inflammation observed in psoriasis patients. Specifically, CCR4^+^ memory T cells, accumulating in peripheral blood, correlate with psoriasis area and severity index (PASI) (7). Additionally, circulating CD8^+^ T cells with a highly differentiated phenotype have been associated with elevated serum levels of C-reactive protein, further linking T cells to systemic inflammation (8, 9).

In psoriasis patients, however, a contribution to cardiovascular disease has been mainly indicated for soluble inflammatory mediators, reaching the circulation and leading to insulin resistance and endothelial dysfunction (10–16). Patients indeed displayed elevated serum level of soluble factors such as vascular endothelial growth factor (VEGF), IL- 8 and TNF-α, and higher serum concentrations of IL-6 correlating with the PASI score (10, 17). Vascular inflammation in psoriasis patients was also clearly evidenced by Mehta and colleagues using (18)F-fluorodeoxyglucose positron emission tomography/computed tomography and was associated with high PASI score (3, 18, 19).

It is now known that chronic inflammation, persistent antigen exposure, and aging can drive memory T cells towards a terminally differentiated or exhausted state (20, 21). These highly to terminally differentiated T cells exhibit altered effector functions, transcriptional regulation, and metabolic imbalances (22). Terminally differentiated/exhausted T cells, characterized by sustained expression of inhibitory receptors such as PD-1, TIM-3, and LAG-3, have been implicated in chronic systemic inflammation and cardiovascular disease (23, 24). In the context of atherosclerosis, T cells expressing markers of exhaustion accumulate within atherosclerotic plaques, where they are thought to contribute to plaque instability and disease progression (25, 26). CXCR3-expressing T cells play a critical role in this process, mediating T cell adhesion and transendothelial migration through interactions with ICAM-1, thus contributing to vascular inflammation (27).

In elderly individuals, T cells with senescent characteristics may shift towards cytotoxic functions, expressing NK cell receptors-like NKG2D, contributing to tissue damage and inflammation (28, 29).

In this light we made the hypothesis that highly differentiated memory CD4^+^ and CD8^+^ T cells, amplified in the psoriatic plaques, can circulate and play a role in the cardiovascular comorbidities associated with psoriasis in a possible synergy with soluble inflammatory mediators. To investigate this hypothesis, we evaluated whether T cells expressing markers of T cell receptor (TCR)-mediated activation, such as 4-1BB or terminal differentiation, PD-1, TIM-3 and NKG2D, accumulate systemically in a mouse model of recurrent psoriasis and we explored their potential role in endothelial dysfunction and vascular inflammation (30, 31).

## Results

### Memory T cell induction in recurrent psoriasis-like inflammation

Recent evidence highlighted the role of T cells in the systemic circulation in the establishment of states of chronic inflammation and tissue damage (26, 32, 33). We therefore, investigate CD4^+^ and CD8^+^ T cell memory phenotype, the expression of chemokine receptors and adhesion molecules, as well as markers of activation and T cell exhaustion in splenocytes from mice with recurrent psoriasis-like inflammation and in control mice. In an initial set of experiments we evaluated whether repeated induction of psoriasis-like inflammation progressively induces the establishment of T cell memory.

**Figure 1:**
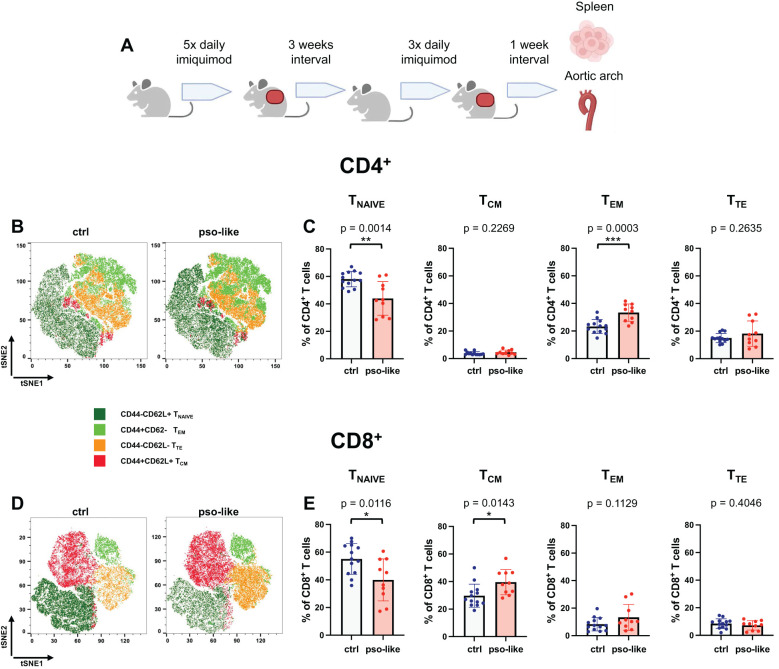
Memory T cells increase in mice with recurrent psoriasis-like inflammation. **(A)** Schematic representation of the experiment. C57BL/6 mice were treated for 5 days with imiquimod on the shaved back skin and ears, and after an interval of three weeks they were treated for three more days. 7 days after the last imiquimod application mice were sacrificed and cells were isolated from the spleen from 4 independent experiments. Original figure created by Biorender.com. Representative tSNE analysis of lymphocyte subpopulations within the CD4^+^ **(B)** and CD8^+^ **(D)** T cells identified by Boolean gating in control mouse and mouse with recurrent psoriasis-like inflammation after down- sampling. Manual gating was performed on t-SNE clusters after annotation. Gated clusters were then overlaid onto the total t-SNE map. The percentage of T_naive_, T_CM_, T_EM_, and T_TE_ in CD4^+^ **(C)** and CD8^+^ **(E)** CD3^+^ splenocytes is reported for the control (crtl, n=13) and recurrent psoriasis-like inflammation groups (pso-like, n=10). The bars represent the mean ± SD. Significance levels of the differences were calculated by Student t-test or Mann-Whitney according on the normality of the distribution. p values < 0.05 were considered significant: *p < 0.05, **p < 0.01, ***p < 0.001.

Imiquimod treatment showed effectiveness in inducing memory T cell response, as showed by a progressive increase in CD44^+^ memory T cells within both CD4^+^ and CD8^+^ populations upon two cycles of imiquimod application (recurrent psoriasis-like inflammation), compared to one cycle of imiquimod application (acute psoriasis-like inflammation) (**Figure 1A**; **Supplementary Figure S1A**). In both compartments, the shift toward CD44^+^ T cells in chronic inflammatory conditions indicates a progressive establishment of T cell memory which may represent a relevant mechanism associated with human disease (**Supplementary Figure S1A**). In animals with recurrent psoriasis-like inflammation, the percentage of CD8^+^ T cells within the CD3^+^ compartment significantly increased compared to the control animals. No significant differences were observed in the percentage of CD4^+^ T cells (**Supplementary Figure S1B**).

We next compared different CD4^+^ and CD8^+^ memory T cell profiles between control (n=13) and recurrent psoriasis-like (n=10) conditions. Both CD4^+^ and CD8^+^ T cells were classified based on CD44 and CD62L expression in T naïve (CD44^-^CD62L^+^), T_CM_ (CD44^+^CD62L^+^), T_EM_ (CD44^+^CD62L^-^), and T_TE_ (CD44^-^CD62L^-^) (**Figures 1B, D**).

A marked decrease in the percentage of naïve T cells was observed in recurrent psoriasis-like inflammation as compared to the control condition in both the CD4^+^ and CD8^+^ compartments (**Figures 1C, E**). In the CD4^+^ T cell compartment, the percentage of T_EM_ was markedly increased in mice with recurrent psoriasis-like inflammation (**Figure 1C**); whereas in the CD8^+^ T cell compartment, T_CM_ cells showed a more pronounced expansion, suggesting an accumulation of long-term memory T cells in the inflammatory setting (**Figure 1E**).

Together these results indicate a marked reduction in naïve CD4^+^ and CD8^+^ T cells in the psoriasis-like condition and an increase in CD8^+^ T_CM_ and CD4^+^ T_EM._

### Memory T Cell phenotype in psoriasis patients compared to healthy subjects

**Figure 2:**
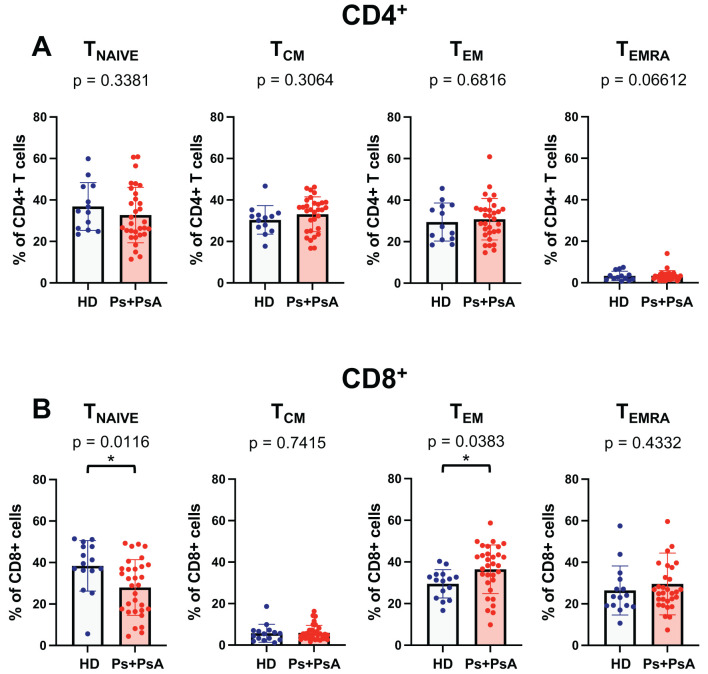
Decrease percentage of naïve T cells and increased percentage of T_EM_ in CD8^+^ T cells from patients with psoriatic disease. The percentage of memory T cells subsets in CD4^+^ **(A)** and CD8^+^ **(B)** subpopulation for a cohort of 15 healthy control subjects and 31 patients with psoriatic disease (psoriasis and psoriatic arthritis) are shown in the figure. Cells were evaluated according to the expression of CD45RA and CCR7 within the lineage marker gate as follows: T_naive_ (CD45RA^+^CCR7^+^), T_CM_ (CD45RA^-^CCR7^+^) T_EM_ (CD45RA^-^CCR7^-^) and T_EMRA_ (CD45RA^+^CCR7^-^). Statistical analysis of the differences was performed by Mann–Whitney test or Student t-test according on the normality of the distribution. p values < 0.05 were considered significant: *p < 0.05.

To compare the results in the mouse model with the human disease, we analyzed our human flow cytometry dataset to specifically define the memory T cell phenotype in patients with psoriatic disease (https://clinicaltrials.gov/ct2/show/NCT03374527) (9, 34). **Figure 2** shows the memory T cell profile in the CD4^+^ and CD8^+^ compartment in healthy donors (HD) and patients with psoriatic disease, including both cutaneous psoriasis and psoriatic arthritis. The subsets analyzed consist of naïve CD4^+^ and CD8^+^ T cells, as well as T_CM_, T_EM_, and terminally differentiated effector memory T cells (T_EMRA_).

Consistent with observations from the mouse model, we found a significant reduction in the percentage of naïve CD8^+^ T cells in psoriasis patients compared to healthy controls (**Figure 2B**). In parallel, psoriasis patients showed a higher percentage of T_EM_ CD8^+^ T cells than healthy donors. However, there were no significant differences in T_EMRA_ between the two groups.

These findings indicate a shift in the CD8^+^ T cell memory phenotype in psoriasis patients, characterized by a marked reduction in naïve T cells and an increase in effector memory. No significant difference was observed in the CD4^+^ compartment (**Figure 2A**).

To further assess whether age differences could have an effect on these observations, we stratified the study cohort by age (< or > 40 years) and sex. Data, shown in **Supplementary Figures S2**, **S3**, indicate no significant differences in memory T cell subsets between age- or sex-stratified groups. This reinforces the evidence that the observed changes in the memory T cell profile are characteristic of psoriasis patients.

### Recurrent psoriasis-like inflammation induced upregulation of CXCR3 on CD4^+^ and CD8^+^ memory T cells and accumulation of CXCR3^+^ 4-1BB^+^ CD4^+^ T cells

**Figure 3:**
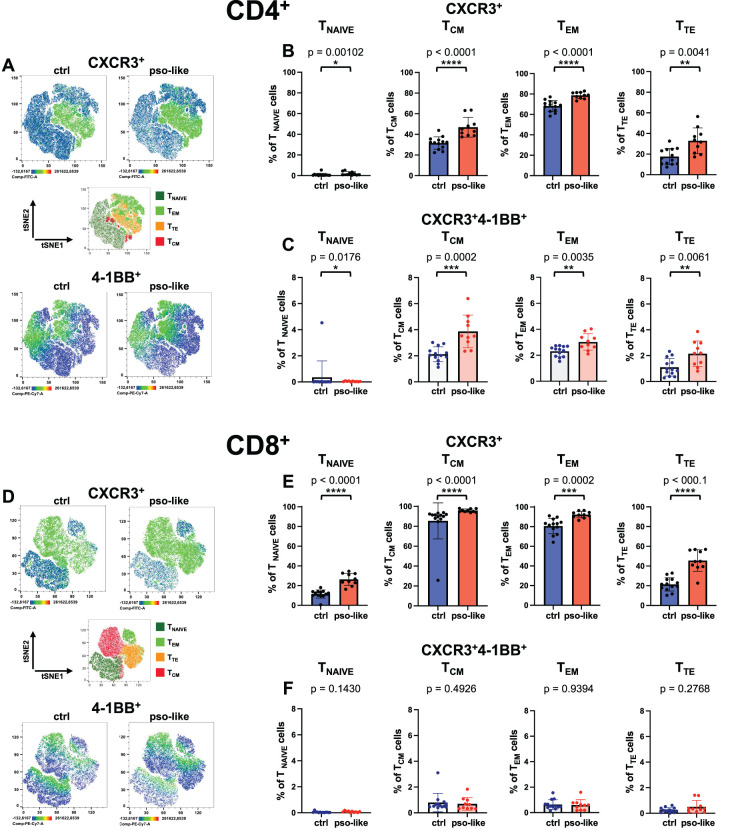
Increased expression of CXCR3 on CD4^+^ and CD8^+^ memory T cells and accumulation of CXCR3^+^ 4-1BB^+^ CD4^+^ T cells in recurrent psoriasis-like conditions. **(A)** Representative Heatmaps display the expression of CXCR3 (upper part of the panel) and 4-1BB (lower part of the panel) on a CD4^+^ t-SNE map identified by Boolean gating from a control mouse and a mouse with recurrent psoriasis-like inflammation. Manual gating of memory T cells (middle part of the panel) was performed on t-SNE clusters after annotation. Percentages of total CXCR3^+^ cells **(B)** and CXCR3^+^4-1BB^+^ cells **(C)** within each CD4^+^ T cell memory subset are shown for the control and psoriasis-like inflammation groups. **(D)** Representative Heatmaps display the expression of CXCR3 (upper part of the panel) and 4-1BB (lower part of the panel) on a CD8^+^ t-SNE map identified by Boolean gating from a control and a psoriasis-like inflammation. Manual gating of memory T cells (middle part of the panel) was performed on t-SNE clusters after annotation. Percentages of total CXCR3^+^ cells **(E)** and CXCR3^+^4-1BB^+^ cells **(F)** within each CD8^+^ T cell memory subset are shown for the control mice (n=13) and mice with recurrent psoriasis-like inflammation (n=10). The bars represent the mean ± SD. Statistical analysis of the differences was performed by Mann–Whitney test or Student t-test according on the normality of the ditribution. p values < 0.05 were considered significant: *p < 0.05, **p < 0.01, ***p < 0.001, ****p<0.0001.

The expression of CXCR3 on CD4^+^ and CD8^+^ T naïve, T_CM,_ T_EM_ and T_TE_ is presented in **Figure 3**, comparing psoriasis-like and control conditions. The data indicates that CXCR3 markedly increased in all subsets of CD4^+^ memory T cells whereas it was not expressed by naïve T cells (**Figures 3A**, upper panel, and **B**). CXCR3 expression was strongly upregulated on T_CM_, T_EM_ and terminal effector CD4^+^ T cells in the recurrent psoriasis-like group.

In the CD8^+^ compartment, CXCR3 expression was also augmented in T_EM_ and T_TE_ cells, with a slight but significant increase also in naive T cells (**Figures 3D**, upper panel, and **E**).

Notably, in the CD4^+^ T cell compartment, the activation marker 4-1BB was upregulated on inflammatory CXCR3^+^CD4^+^ T cells in all memory subsets in psoriasis-like conditions compared to the control group, with a more pronounced increase in CD4^+^ T_CM_ and T_TE_ cells (**Figure 3C**) (30). By contrast, the 4-1BB^+^ cells were not increased on CXCR3^+^ CD8^+^ T cells in psoriasis-like conditions (**Figure 3F**).

This data suggests that highly differentiated, potentially pro-inflammatory T cells with enhanced capability to be recruited at inflamed sites are augmented in psoriasis-like conditions.

### Increased percentage of TIM-3^+^PD-1^+^ CD4^+^ T cell effectors in recurrent psoriasis-like inflammation

**Figure 4:**
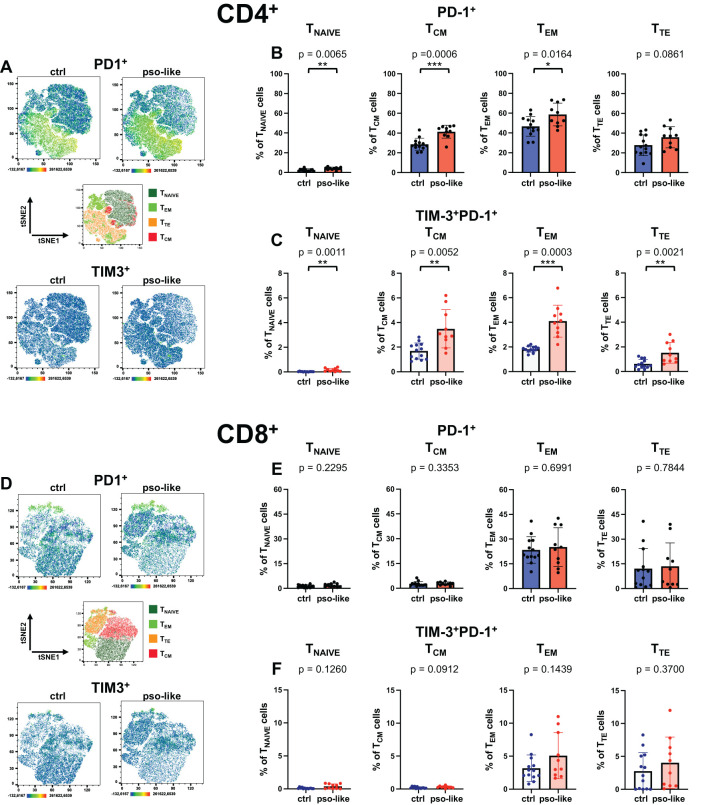
Increased percentage of TIM-3^+^PD-1^+^ CD4^+^ T cells in recurrent psoriasis-like inflammation. **(A)** Representative Heatmaps display the expression of PD-1 (upper part of the panel) and TIM-3 (lower part of the panel) on a CD4^+^ t-SNE map identified by Boolean gating from control mouse and mouse with recurrent psoriasis-like inflammation. Manual gating of memory T cells (middle part of the panel) was performed on t-SNE clusters after annotation. Percentages of total PD-1^+^ cells **(B)** and PD-1^+^TIM-3^+^ cells **(C)** within each CD4^+^ T cell memory subset are shown for the control and psoriasis-like groups. **(D)** Heatmaps display the expression of PD-1 (upper part of the panel) and TIM-3 (lower part of the panel) on a CD8^+^ t-SNE map identified by Boolean gating from control mouse and mouse with recurrent psoriasis-like inflammation. Manual gating of memory T cells (middle part of the panel) was performed on t-SNE clusters after annotation. Percentages of total PD-1^+^ cells **(E)** and PD-1^+^TIM-3^+^ cells **(F)** within each CD8^+^ T cell memory subset are shown for the control mice (n=13) and mice with recurrent psoriasis-like inflammation (n=10). The bars represent the mean ± SD. Statistical analysis of the differences was performed by Mann–Whitney test or Student t-test according on the normality of the distribution. p values < 0.05 were considered significant: *p < 0.05, **p < 0.01, ***p < 0.001.

We next want to specifically analyze the markers of terminal differentiation/exhaustion, namely PD-1 and TIM-3 (**Figures 4A, B, D, E**). We found that inhibitory checkpoint PD-1 progressively increased its expression in CD4^+^ T cells, from naive to T_EM_ and decreased in terminally differentiated T cells. In all subsets PD-1 expression was enhanced by recurrent psoriasis (**Figures 4A, B**). The analysis revealed an increase in the percentage of TIM-3^+^ PD-1^+^ in CD4^+^ T cells across the memory subsets with T_EM_ cells increasing the percentage of exhausted T cells to the highest-level (**Figure 4C**). This dual expression highlights a terminal/exhausted phenotype in these cells, indicative of a low-grade chronic activation state (35).

In CD8^+^ T cells, PD-1 expression was not significantly increased (**Figure 4D**, upper panel; **Figure 4E**), nor was there a significant change in the percentage of TIM-3^+^PD-1^+^ cells within individual memory subsets (**Figure 4F**). Across the different subsets, we did not detect any expression of NKG2D, either in the control group or in recurrent psoriasis-like inflammation (data not shown). We therefore analyzed the expression of CD69, showing an increase of TIM-3^+^CD69^+^ in CD4^+^ T_CM_ and T_EM_ cells, with a similar trend in T_TE_ (**Supplementary Figure S4A**).

### Systemic and vascular inflammation in recurrent psoriasis-like conditions

**Figure 5:**
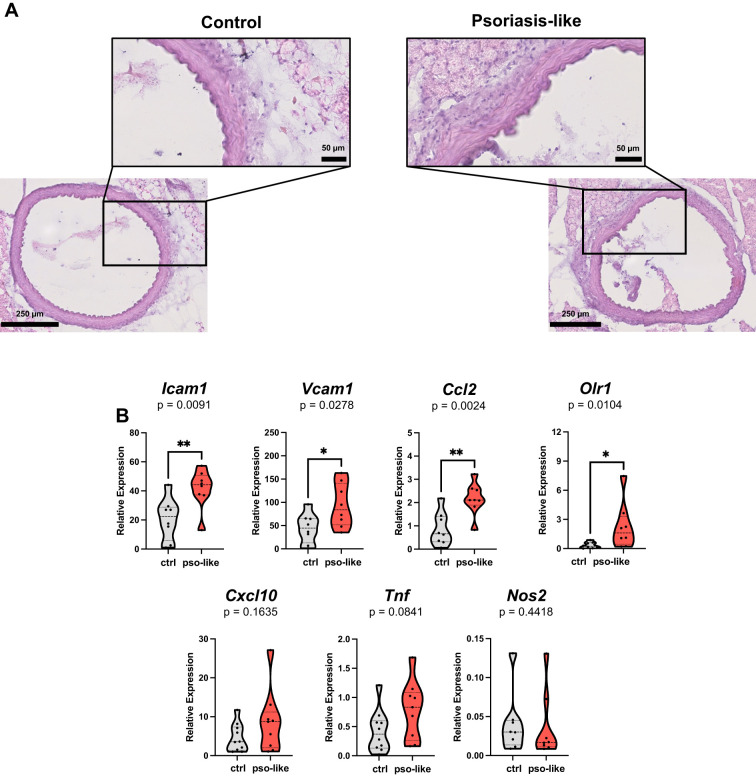
Increased expression of endothelial dysfunction and inflammation signature genes in aorta samples from mice with psoriasis-like inflammation. **(A)** Hematoxylin and eosin slide of aorta from control mice (left part of the panel) and mice with recurrent psoriasis like inflammation (right part of the panel). Scale bars are reported on the images. **(B)** Gene expression analysis by qRT-PCR in aorta samples from 12 control mice and 10 mice with recurrent psoriasis-like inflammation. Relative expressions were calculated using the 2^−ΔCT^ formula and *Rpl13A* as a reference gene. Violin plots showing the relative expression for each gene in the two conditions are reported in the figure. Statistical analysis of the differences was performed by Mann–Whitney test or Student t-test according to the normality of the distribution. p values < 0.05 were considered significant: *p < 0.05, **p < 0.01.

To explore the putative link between systemic T cell activation and vascular inflammation we analyzed in aorta samples histological and molecular signs of endothelial dysfunction. Aorta samples were collected for histological analysis or RNA extraction and analysis of the expression of signature genes related to endothelial activation and vascular inflammation. Histological analysis on sections from mice with recurrent psoriasis-like inflammation reveals discontinuous endothelial layer and impaired organization of elastic and muscular fibers in *tunica media*. Immune cell infiltration was mainly observed in the connective tissue around the vessel (**Figure 5A**).

Consistently gene expression analysis in aorta samples showed elevated markers of endothelial activation, such as *Icam1* and *Vcam1* in psoriasis-like condition. In parallel, the expression of the vascular inflammation marker *Ccl2*, was markedly upregulated (**Figure 5B**). At the time of aorta collection, we did not find any increase in the level of IL-6 in the serum of mice with recurrent psoriasis-like inflammation compared to control, whereas IL-6 was significantly increased at day 3 during the first imiquimod treatment as previously reported (**Supplementary Figure S5**) (36). This indicates that the induction of ICAM-1 and endothelial dysfunction cannot be ascribed to a direct effect of IL-6 but it is rather a long-lasting change.

Notably, in recurrent psoriasis-like conditions, we observed an increase in the expression of *Olr1*, a gene associated with atherosclerosis (37, 38). Importantly, we also found a trend towards an increase in the expression of *Cxcl10* which is the ligand of CXCR3 and can represent the link to T cells.

**Figure 6:**
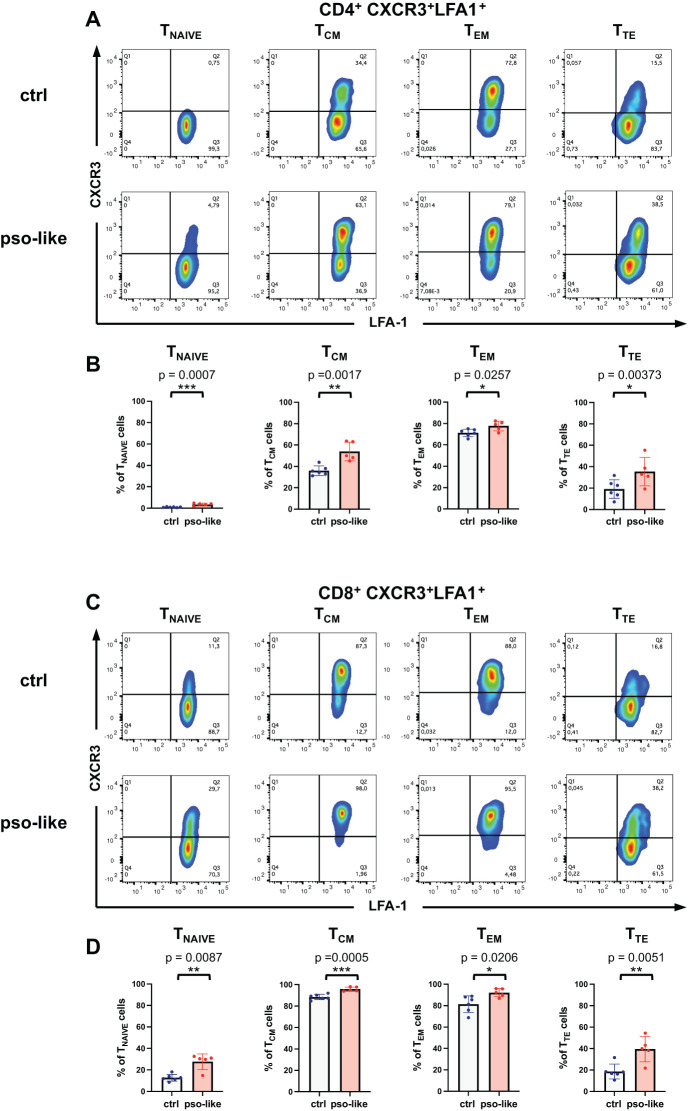
Increased expression of LFA-1^+^ CXCR3^+^ in CD4^+^ and CD8^+^ T cell subsets. **(A)** Representative dot plots showing the LFA-1 and CXCR3 expression within each CD4^+^ T cell memory subsets are presented for control mice (n=6) and mice with recurrent psoriasis-like inflammation (n=5). **(B)** The percentage of LFA-1^+^CXCR3^+^ gated cells within each CD4^+^ memory subset in control and recurrent psoriasis-like group are reported in the figure. Statistical analysis of the differences was performed by Student t-test according to the normality of the distribution, p-values ≤ 0.05 were considered significant. **(C)** Representative dot plots showing the LFA-1 and CXCR3 expression within each CD8^+^ T cell memory subsets are presented for control mice (n=6) and mice with recurrent psoriasis-like inflammation (n=5) in the figure. **(D)** The percentage of LFA-1^+^CXCR3^+^ gated cells within each CD8^+^ memory subset in the control and recurrent psoriasis-like group are reported in the figure. Statistical analysis of the differences was performed by Mann-Whitney test or Student t-test according to the normality of the distribution. p values < 0.05 were considered significant: *p < 0.05, **p < 0.01, ***p < 0.001.

To further explore this putative link, we analyzed the expression of LFA-1 ligand of ICAM-1 on memory T cells with a proinflammatory effector phenotype. We observed a significant increase in LFA-1^+^CXCR3^+^ cells in all CD4^+^ memory subsets with a marked increase in the T_TE_ cells (**Figures 6A, B**). CXCR3^+^LFA-1^+^ cells also increased in all subsets of CD8^+^ memory T cells (**Figures 6C, D**). The difference between psoriasis-like and control conditions became even more pronounced in terminally differentiated CD8^+^ T_TE_ cells.

## Discussion

Our study investigates the systemic impact of recurrent psoriasis-like inflammation on T cell differentiation, activation and vascular inflammation, using a murine model and integrating findings from human psoriatic patients. The results reveal that repeated induction of psoriasis-like inflammation results in a significant expansion of memory T cells, with a reduction in naïve CD4^+^ and CD8^+^ T cell populations. Notably, both T_CM_ and T_EM_ cells were significantly increased, with CD4^+^ T_EM_ cells showing heightened activation markers. This aligns with previous studies indicating that chronic antigen exposure can drive memory T cells into terminally differentiated states, leading to pro-inflammatory phenotypes that may propagate systemic inflammation (31, 39, 40).

The upregulation of exhaustion markers TIM-3 and PD-1 on CD4^+^ T cells in recurrent psoriasis-like conditions indicates an exhausted phenotype linked to chronic activation which has been previously implicated in chronic inflammatory states (23, 24, 41). The sustained presence of these terminally differentiated cells equipped with proinflammatory molecules can promote low-grade inflammation with a potential involvement in the generation of an atherogenic environment (29). Histological analysis indeed indicates increased inflammation-mediated remodeling of the vessel wall, and increase infiltration of immune cells, especially in *adventitia*, in psoriasis-like inflammation.

The role of CXCR3 and LFA-1 in mediating vascular inflammation is noteworthy. A previous study by Ngwenyama et al. showed upregulation of LFA-1, that can interact with ICAM-1 on endothelial cells, enhancing CD4^+^ T cell adhesion and migration (27). Therefore, increased CXCR3 expression on T cells within the spleens in recurrent psoriasis-like conditions provides an important mechanistic insight. The observation that CXCR3^+^ LFA-1^+^ T cells increased systemically in psoriasis-like conditions indicates that chronic skin inflammation may induce T cells with enhanced migratory capacity. In parallel, upregulation of *Icam1, Vcam1*, and *Ccl2* in aortic tissues from psoriatic mice was observed, together with a clear trend towards an increase in the expression of *Cxcl10* chemokine, ligand of CXCR3. These vascular tissue changes are likely initiated by increased systemic inflammatory burden and indicate a mechanistic link between soluble mediators of inflammation, vascular inflammation and highly differentiated T cell recruitment. The vascular tissue changes, however, cannot be ascribed only to the increase of soluble inflammatory molecules, as the serum levels of IL-6 were not increased at the time of aorta collection, indicating long lasting-modifications possibly initiated by an earlier cytokine storm.

A key role could be played by MCP-1/CCL-2 monocyte chemotactic factor upregulated in recurrent psoriatic-like conditions. Myeloid cells in turn can express CXCL10 linking to T cells. MCP-1/CCL2 is produced by smooth muscle and endothelial cells upon exposure to soluble inflammatory molecules and is known to contribute to chronic inflammation in atherosclerotic plaques (42). In support of the generation of an atherogenic environment we also observed a significantly increased expression of *Olr1 (Lox-1)* encoding oxidized low-density lipoprotein (oxLDL) receptor (LOX-1) a transmembrane protein expressed by endothelial cells, smooth muscle cells, cardiomyocytes and macrophages. Activation of LOX-1 by oxLDL is involved in atherosclerosis and, in animal models, its pharmacological inhibition resulted in plaque regression (37, 38). Therefore, changes in the vascular tissues, likely initiated by the soluble inflammatory mediators such as IL-6 (12) could favor the establishment of a proatherogenic environment with upregulated ICAM-1 and CXCL10, promoting the interaction with highly differentiated T cells expressing LFA-1 and CXCR3.

In conclusion, this study provides insights into the mechanistic link between systemic T cell activation and vascular inflammation, enhancing the understanding of cardiovascular risks associated with psoriasis.

## Methods

### Experimental design

The aim of this study is to evaluate the impact of repeated induction of psoriasis-like inflammation on the generation of CD4^+^ and CD8^+^ T cell memory and on their activation state, functional properties and differentiation stage in the spleen of C57BL/6 mice.

In the mouse models of recurrent psoriasis-like inflammation, imiquimod application was given in two phases: one first round of applications for 5 days followed by 3 weeks interval and a second round of applications for three days according to the scheme described by Ramirez-Valle and colleagues (43) and reported in **Figure 1A**. One week after the last imiquimod application, splenocytes were analyzed for the memory T cell phenotype, for the presence of markers of terminal differentiation, of chemokine receptors and activation markers. We also analyzed NKG2D that was reported to be expressed by terminally differentiated CD4^+^ T cells in elderly people (44).

### Mice

All procedures involving mice, and their care were performed in conformity with the ethical principles and EU Directive 2010/63/EU for animal experiments guidelines. The project was approved by the Italian Ministry of Health (Aut. n. 364/2019-PR and n.1067/2023-PR) and all experiments were performed in accordance with the project and with the local ethical committee. C57BL/6 mice were housed in pathogen-free conditions with food and water available *ad libitum*. 6–12 weeks female and male mice were used for the experiments. Shaving and imiquimod applications were performed under brief inhalation anesthesia with 2% isoflurane. At the end of the experiment, the mice were euthanized by cervical dislocation.

### Recurrent imiquimod-induced psoriasis-like inflammation

Wild-type C57BL/6 mice were used in the experiments. For the induction of psoriasis-like inflammation, each mouse was treated with imiquimod (ALDARA 50 mg/day) for 5 consecutive days on the shaved back and ears. After 3 weeks from the last treatment, the mice, which were fully recovered, were treated once more for 3 days according to the schedule first described by Ramirez-Valles et al. (36, 43). One week after the end of the second treatment, mice were sacrificed, spleen and aorta were collected for flow cytometry and gene expression analysis or histology, respectively (**Figure 1A**).

### Spleen collection and purification

Following euthanasia, spleens were excised and placed in sterile cell strainers over a Petri dish containing 4 mL of medium supplemented with 10% FBS (Euroclone; Milan, Italy). Each spleen was manually dissociated until the capsule was emptied. The resulting splenocyte suspension was washed via centrifugation at 1300 rpm for 10 minutes and subsequently lysed by adding 4 mL of cold, sterile 1X lysis buffer (composed by 8.26 g NH4Cl, 1 g KHCO3, and 0.037 g EDTA in 100 mL Milli-Q water). After a 5-minute incubation on ice, the suspension was diluted with medium and subjected to two additional washes by centrifugation. The final cell pellet was resuspended in an appropriate volume of medium for subsequent experimental use.

### Aorta collection and storage

The mouse was placed in the supine position on a dissection board, and its limbs were secured. Using sterile surgical scissors, a longitudinal incision was then made in the thoracic cavity, extending through the diaphragm to the base of the heart, revealing the thoracic aorta and its branching vessels. The aorta is gently separated and excised from surrounding tissues using fine-tipped forceps and micro scissors. The excised aorta was then transferred into cryovials and stored at -80°C for molecular analyses (45). Alternatively, aorta samples were embedded in optimal cutting temperature freezing media (Bio-Optica). Sections (20 μm), were cut on a cryostat, adhered to a Superfrost™ Plus Microscope Slides (ThermoFisher Scientific, Monza MB, Italy) and fixed with acetone.

### Hematoxylin and eosin staining

Aorta sections were stained with Meyer’s hematoxylin solution for 2 minutes, followed by a 5-minute wash in warm running tap water. Sections were stained with Eosin Y solution for 1 minute, washed in warm running tap water for 5 minutes, rinsed in distilled water, and finally dehydrated through a series of passages in 95% and absolute ethanol. After dehydration, stained slides were cleared in xylene and mounted with Eukitt. Images were acquired with the NanoZoomer (Hamamatsu, Shizuoka, Japan).

### Phenotype analysis of splenocytes

Flow cytometric immunophenotyping of single-cell suspensions obtained from smashed spleen was conducted using standard protocols and combinations of pre-titered fluorochrome-conjugated antibodies (Miltenyi Biotech GmbH, Bergisch Gladbach, Germany) in the dark for 30 min at RT in PBS. A combination of the following fluorochrome-conjugated antibodies was used: CD3 (REA641), CD4 (REA604), CD8a (53-6.7), CD44 (REA644), CD62L (REA828), CD69 (REA937), CD314 (NKG2D; CX5), CD183 (CXCR3; REA724), CD137 (4-1BB; REA936), CD366 (TIM-3; REA602), CD279 (PD-1; REA802) and LFA (REA880). Live and dead cell discrimination was performed by incubation of the cells with Aqua™ (eBioscience, San Diego, CA, USA) viable dye.

Cells were then fixed with 2% paraformaldehyde for 30 minutes at 4°C, then washed with PBS and acquired using FACSAria III Cytometer (BD Biosciences, Franklin Lakes, NJ, USA). Data was analyzed with the FlowJo™ Software v10.10 (BD Biosciences, Ashland, OR, USA). The gating strategy is reported in **Supplementary Figure S6**.

Fluorescence minus one (FMO) controls including all the fluorophores being used in the panel except for the ones of interest, were used to evaluate non-specific fluorescence as previously described (46).

### RNA extraction and real-time PCR

Total RNA was extracted from aorta samples collected by individual mice of each experimental group using TRIzol (Invitrogen, Milan, Italy) according to the manufacturer’s instructions. After centrifugation, the aqueous phase containing RNA was collected and passed through RNeasy mini kit columns (Qiagen, Hilden, Germany). RNA quantity and quality after purification was assessed using the Nanodrop ND-100 Spectrophotometer (Nanodrop Technologies, Wilmington, DE), with a 260:280 ratio of >1.8.

An equal amount of RNA (500 ng/sample) from aorta samples was retrotranscribed to complementary DNA using SuperScript™ III First-Strand Synthesis SuperMix (ThermoFisher) as described by the manufacturer. Retrotranscribed samples were used in qRT-PCR reaction using PerfeCTa SYBR^®^ Green SuperMix Reagent (QuantaBio) with 200 nM of forward and reverse primers. The primers for *Icam1*, *Vcam1*, *Ccl2*, *Olr1*, *Tnf*, *Cxcl10*, and *Nos2* are reported in **Supplementary Table S1**. qRT-PCR was performed by using 7900 HT Real-Time PCR System (Applied Biosystems). The relative gene expression level was calculated using the 2^−ΔCT^ formula, in which the ΔCT represents the difference between the threshold cycle of the target gene and the reference gene, Ribosomal Protein L13a (*Rpl13a*), for each sample analyzed.

### Statistical analysis

Data presented as means were compared by either unpaired parametric t-tests or nonparametric tests, depending on the normality of the distribution, which was evaluated by the Shapiro–Wilk test. Data were expressed and plotted as mean ± SD values. Sample sizes for each experimental condition were provided in the figure legends. All p values were calculated using GraphPad Prism (GraphPad Software, La Jolla, CA, USA), and tests were considered significant when the p values were lower than 0.05.

## Data Availability

The raw data supporting the conclusions of this article will be made available by the authors, without undue reservation.
