# Supplementary material for: Highly differentiated T cells link systemic and vascular inflammation in a mouse model of recurrent psoriasis
Source: Front Immunol. 2025 Jun 17;16:1574455. doi: 10.3389/fimmu.2025.1574455 (PMC12208849; doi:10.3389/fimmu.2025.1574455)
Supplement: Supplementary file 2 [file DataSheet2.pdf]

Highly differentiated T cells link systemic and vascular inflammation in a mouse model of recurrent psoriasis

[*1]Fabio Casciano, [*2]Paolo Severi, [*3]Laura Marongiu, [1]Anna Caproni, [2]Chiara Terranova, [2]Alex Spitilli, [4]Davide Ferrari, [5]Chiara Ruzza, [1]Peggy C.R. Marconi, [6,7]Paola Rizzo, [3]Francesca Granucci, [2]Paola Secchiero and [2+]Eva Reali

Front. Immunol. doi: 10.3389/fimmu.2025.1574455

* The authors share the first author position
[1]Department of Environmental and Prevention Sciences and LTTA Centre, University of Ferrara, Ferrara, Italy
[2]Department of Translational Medicine, University of Ferrara, Ferrara, Italy
[3]Department of Biotechnology and Biosciences, University of Milano-Bicocca, Milan, Italy
[4]Department of Life Sciences and Biotechnology, University of Ferrara, Ferrara, Italy
[5]Department of Neurosciences and Rehabilitation, University of Ferrara, Ferrara, Italy
[6]Department of Translational Medicine and LTTA Centre, University of Ferrara, Ferrara, Italy
[7]Maria Cecilia Hospital, GVM Care & Research, Cotignola, Italy

+ Address for Correspondence:
Eva Reali Ph.D
Department of Translational Medicine, via Luigi Borsari 46, 44121, Ferrara Italy
Email: eva.reali@unife.it

Supplementary Information

Supplementary materials and methods:

ELISA assay for IL-6 quantification in mouse serum:
Mouse serum was collected from mice on day 3 during the first imiquimod treatment and at the time of the sacrifice, one week after the end of the second treatment. Serum was diluted from 1:3 to 1:10 in serum dilution buffer and quantified using the IL-6 Elisa Kit PeproTech *(cat. n.* 900-T50 + HRP Conjugate) according to the manufacturer instruction.

Supplemental Table 1

Primer name	Primer Sequence
Rpl13A - F	AGCCCAGGGTGCTTTGCGG
Rpl13A - R	GCGCCATGGCTGCCTCCTATAC
Icam1 - F	CACGTGCTGTATGGTCCTCG
Icam1 - R	TAGGAGATGGGTTCCCCCAG
Vcam1 - F	ATGTCAACGTTGCCCCCAA
Vcam1 - R	GCTGTCTGCTCCACAGGATT
Ccl2 - F	AGCTGTAGTTTTTGTCACCAAGC
Ccl2 - R	GACCTTAGGGCAGATGCAGT
Olr1 - F	TGCAAACTTTTCAGGTCCTTGT
Olr1 - R	AACTGGCCACCCAAAGATTG
Cxcl10 - F	ATGACGGGCCAGTGAGAATG
Cxcl10 - R	ATTCTTTTTCATCGTGGCAATGA
Tnf - F	GCACAGAAAGCATGACCCG
Tnf - R	GCCCCCCATCTTTTGGG
Nos2 - F	AGTCAACTGCAAGAGAACGGA
Nos2 - R	GAAGAGAAACTTCCAGGGGCA

Supplemental Table 1. Primers

Supplemental Figure 1

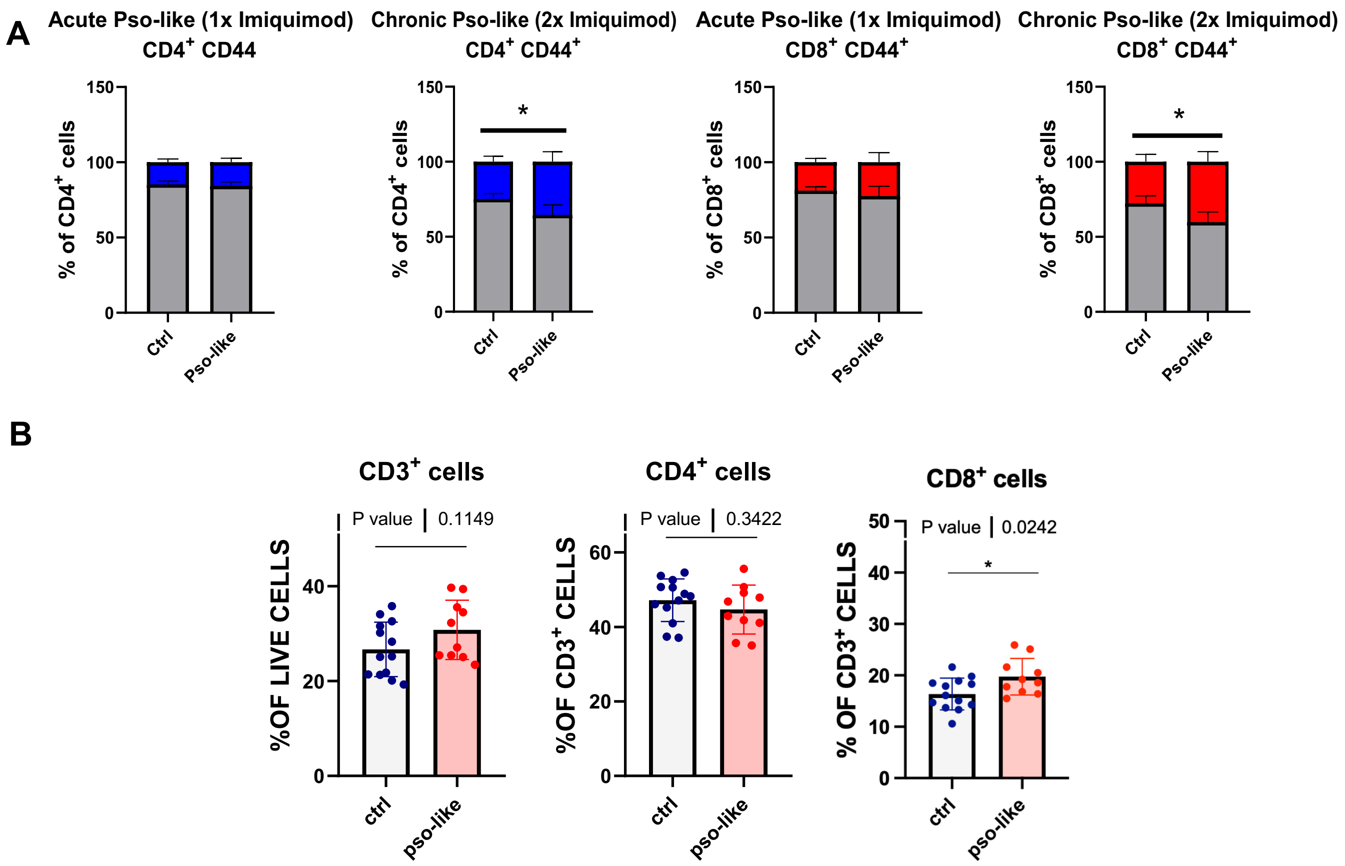

Progressive accumulation of CD44+ memory T cells in chronic pso-like condition compared to acute pso-like and increased CD8+ T cells frequency in recurrent pso-like.

(A) Percentage of CD44+ memory T cells in CD4+ and CD8+ T cells from C57BL/6 mice (n=4/group) upon induction of acute (1 round of imiquimod application) and chronic (2 rounds of imiquimod applications) psoriasis-like inflammation and in untreated control conditions.
Stacked bar represents the mean ± SD of the percentage of CD44+ (red or blue bars) and CD44- (gray bars) cells in CD4+ and CD8+ gated cells. Statistical analysis of the differences was performed by either Student's t-test or Mann-Whitney test depending on normality of the data distribution (p value ≤ 0.05).

(B) The percentage of CD3+ cells in the viable lymphocyte gate and the percentages CD4+ and CD8+ T cells in the CD3+ gated cells, was compared between control (n=13) and recurrent psoriasis-like conditions (pso-like) (n=10). Bar represents the mean ± SD of the percentage of the different subpopulation in control and pso-like condition. Statistical analysis of the differences was performed by either Student's t-test or Mann-Whitney test depending on normality of the data distribution (p value ≤ 0.05).

Supplemental Figure 2

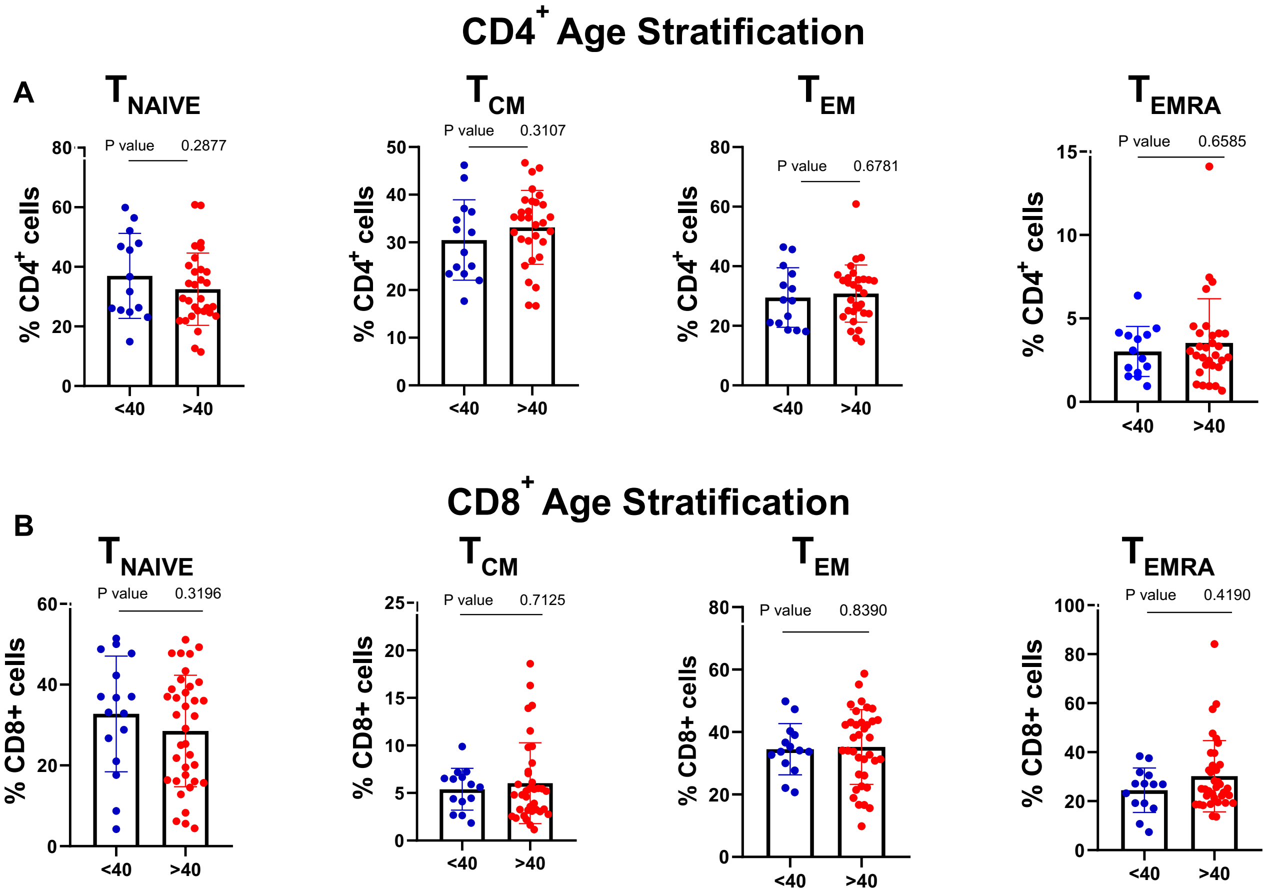

Percentages of the individual memory T cells subset in CD4+ and CD8+ T cells from all subjects of the cohort divided on the age-basis.

Percentages of CD4+ (A) and CD8+ (B) memory T cell subsets: T_{NAIVE} (CD45RA+CCR7+), T_{CM} (CD45RA-CCR7+) T_{EM} (CD45RA-CCR7-) and T_{EMRA} (CD45RA+CCR7-) in human subjects comprising patients with psoriatic disease (psoriasis and psoriatic arthritis) (n=31) and healthy subjects (n=15) of the same cohorts. The subjects were divided on the age basis (<40 years and >40 years). Statistical analysis of the differences between the two age groups was performed by either Student's t-test or Mann-Whitney test depending on normality of distribution (p value ≤ 0.05).

Supplemental Figure 3

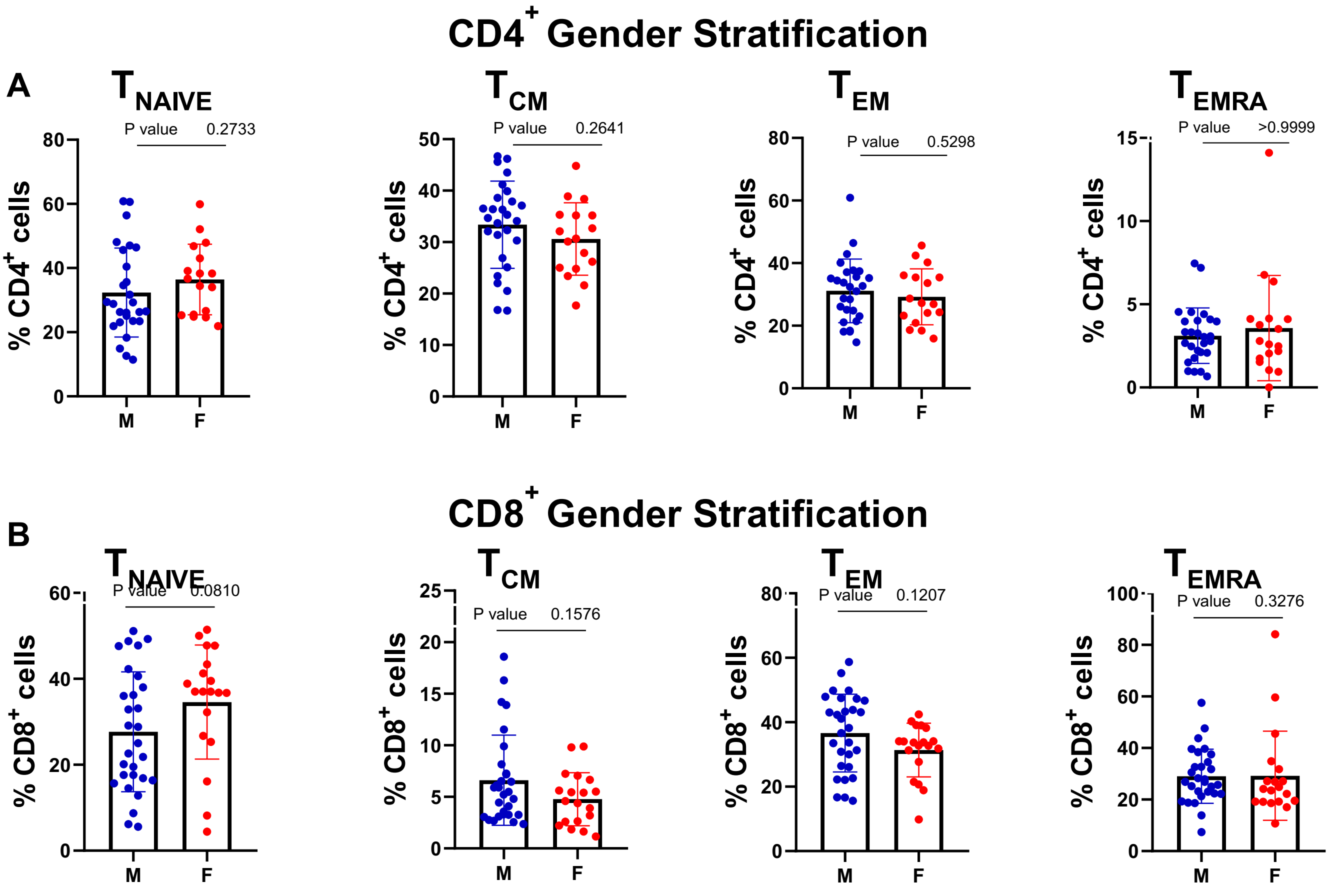

Percentages of the individual memory T cells subset in CD4+ and CD8+ T cells from all subjects of the cohort divided on the sex-basis.

Percentages of CD4+ (A) and CD8+ (B) memory T cells subsets: T_{NAIVE}(CD45RA+CCR7+), T_{CM} (CD45RA-CCR7+) T_{EM} (CD45RA-CCR7-) and T_{EMRA} (CD45RA+CCR7-) in male and female subjects from the same cohort comprising patients with psoriatic disease (psoriasis and psoriatic arthritis) (n=31) and healthy subjects (n=15). The subjects were divided on the sex-basis (male and female groups). Statistical analysis of the differences between the male and female groups was performed by either Student's t-test or Mann-Whitney test according to the normality of distribution and were considered significant for p values ≤ 0.05.

Highly differentiated T cells link systemic and vascular inflammation in a mouse model of recurrent psoriasis.
Casciano F., Severi P., Marongiu L. et al. Front. Immunol. doi: 10.3389/fimmu.2025.1574455

Supplemental Figure 4

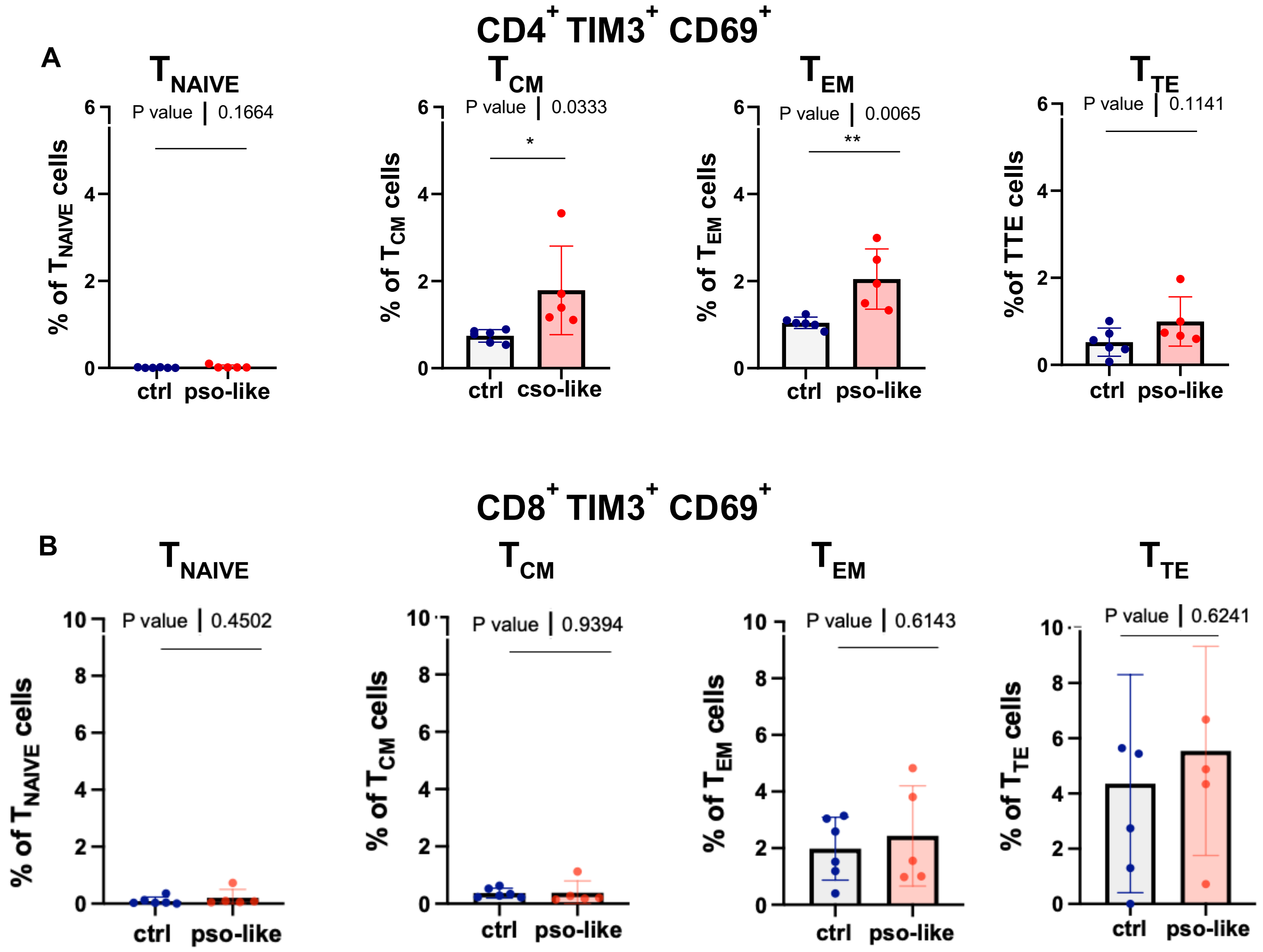

Increased of TIM3+CD69+ on CD4+ memory T cells subsets

Quantitative analysis of the percentage of CD69+ TIM3+ on CD4+ (A) and CD8+ (B) memory T cells subsets: TNAIVE, TCM, TEM, TTE, in recurrent psoriasis-like (n=5) and control (n=6) conditions. Bars represent the mean ± SD of the percentages of TIM3+CD69+ cells in each subpopulation. Significance levels of the differences between groups were calculated by Student t -test or Mann–Whitney test according to the normality of the distribution and were considered significant for p values ≤ 0.05.

Highly differentiated T cells link systemic and vascular inflammation in a mouse model of recurrent psoriasis.
Casciano F., Severi P., Marongiu L. et al. Front. Immunol. doi: 10.3389/fimmu.2025.1574455

Supplemental Figure 5

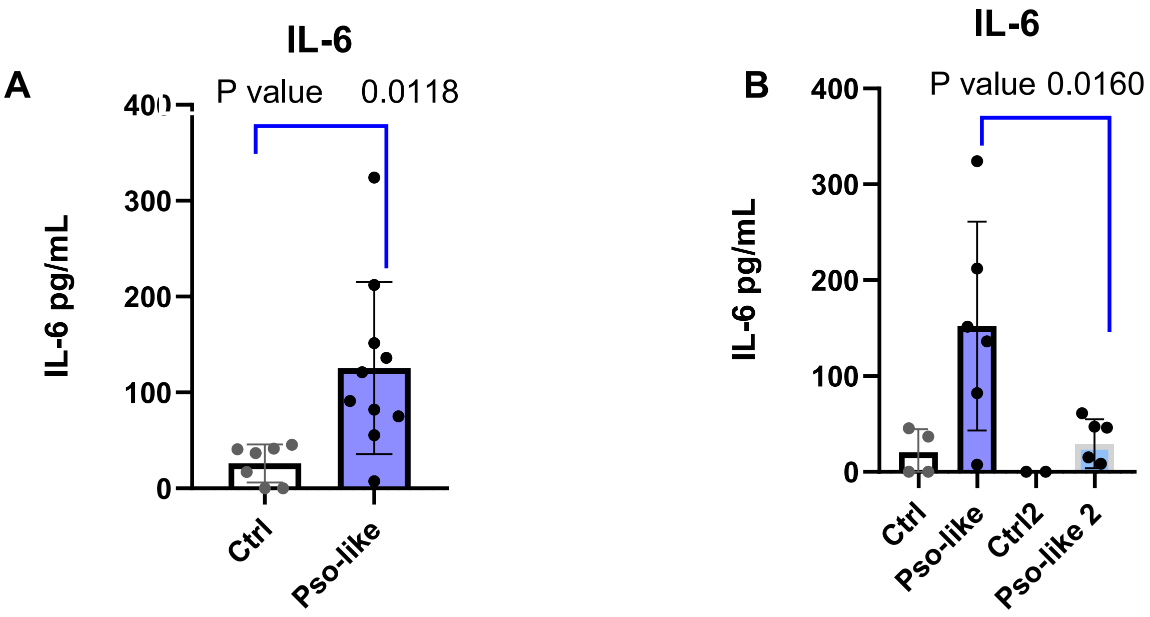

IL-6 Concentration Measurement

Serum samples were collected on day 3 during the first imiquimod treatment and at the time of sacrifice, which was one week after the second treatment. Serum was diluted from a ratio of 1:3 to 1:10 in a serum dilution buffer. IL-6 levels were quantified by ELISA assay.

(A) Comparison between Control and Imiquimod-Treated Group (day 3 of the first treatment): IL-6 concentration was compared between the control group and the imiquimod-treated group on day 3 of the first treatment. Statistical Analysis: Unpaired t-test was used for the comparison. p-value of ≤ 0.05 was considered significant.
(B) Comparison between first and second Imiquimod Treatment: IL-6 concentrations were compared within individual mice between the first and second imiquimod treatment.
Paired t-test was used for comparing the paired data from the same subjects. p-value of ≤ 0.05 was considered significant.

Highly differentiated T cells link systemic and vascular inflammation in a mouse model of recurrent psoriasis.
Casciano F., Severi P., Marongiu L. et al. Front. Immunol. doi: 10.3389/fimmu.2025.1574455

Supplemental Figure 6

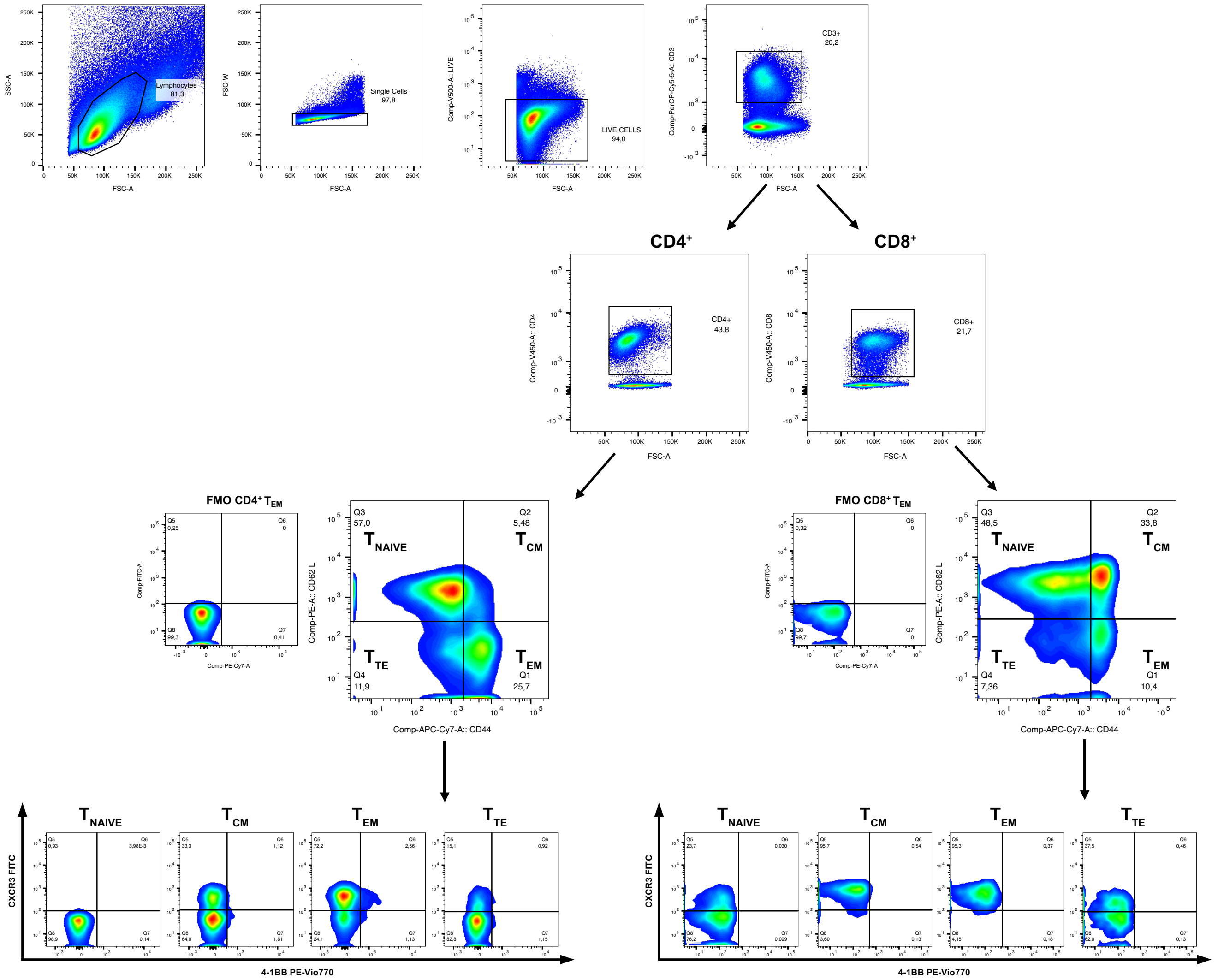

Gating strategy for the analysis of mouse memory T cells.

The gating strategy used to characterize T CD4+ and CD8+ cells in the spleen is shown in the figure. Splenocytes from one representative mouse, were stained with CD3, CD4, CD8, CD62L, CD44, CD137 (4-1BB) and CXCR3 fluorochrome-conjugated antibodies and analyzed by flow cytometry. The representative analysis shows the staining of CD4+and CD8+ T cells for the memory phenotype CD44/CD62L. Negative controls were performed using Fluorescence minus one (FMO) staining. The axis scales for fluorescence are reported as log; the axis scales for SSC, FSC are reported as linear.
